# Clinicians’ and patients’ views of metrics of change derived from patient reported outcome measures (PROMs) for comparing providers’ performance of surgery

**DOI:** 10.1186/1472-6963-12-171

**Published:** 2012-06-21

**Authors:** Zoe Hildon, Jenny Neuburger, Dominique Allwood, Jan van der Meulen, Nick Black

**Affiliations:** 1 Department of Health Services Research & Policy, London School of Hygiene & Tropical Medicine, 15-17 Tavistock Place, London, WC1H 9SH, UK

## Abstract

**Background:**

Patient reported outcome measures (PROMs) are increasingly being used to compare the performance of health care providers. Our objectives were to determine the relative frequency of use of different metrics that can be derived from PROMs, explore clinicians’ and patients’ views of the options available, and make recommendations.

**Methods:**

First a rapid review of the literature on metrics derived from two generic (EQ-5D and EQ-VAS) and three disease-specific (Oxford Hip Score; Oxford Knee Score; Aberdeen Varicose Vein Questionnaire) PROMs was conducted. Next, the findings of the literature review were mapped onto our typology of metrics to determine their relative frequency of use, Finally, seven group meetings with surgical clinicians (n = 107) and six focus groups with patients (n = 45) were held which were audio-taped, transcribed and analysed thematically.

**Results:**

Only nine studies (9.3% of included papers) used metrics for comparing providers. These were derived from using either the follow-up PROM score (n = 3) or the change in score as an outcome (n = 5), both adjusted for pre-intervention score. There were no recorded uses of the proportion reaching a specified (‘good’) threshold and only two studies used the proportion reaching a minimally important difference (MID).

Surgical clinicians wanted multiple outcomes, with most support expressed for the mean change in score, perceiving it to be more interpretable; there was also some support for the MID. For patients it was apparent that rather than the science behind these measures, the most important aspects were the use of language that would make the metrics personally meaningful and linking the metric to a familiar scale.

**Conclusions:**

For clinicians the recommended metrics are the mean change in score and the proportion achieving a MID, both adjusted for pre-intervention score. Both need to be clearly described and explained. For patients we recommend the proportion achieving a MID or proportion achieving a significant improvement in hip function, both adjusted for pre-intervention score.

## Background

There is increasing use of patient reported outcome measures (PROMs) to assess and compare the performance of health care providers. Since April 2009, their use for measuring the outcome of four common elective operations (hip and knee replacement, hernia repair, varicose vein surgery), using before and after data, has been mandatory in the NHS in England. [1,2] Their use is likely to expand in the future. It is envisaged that PROMs will help improve the quality of care through several mechanisms: enhancing patient choice, encouraging clinicians and managers to review patient care, informing commissioners' decisions and providing information for regulators. [3-5]

Although PROMs have been subjected to extensive testing of their measurement properties, less attention has been paid to the range of metrics that can be derived from before and after PROMs data. Our aim was to recommend the most appropriate metrics for comparison of the providers of surgery for presentation to clinicians and to patients. To achieve this, the objectives were: to identify the different metrics that can be derived from PROMs; to assess the relative frequency with which these have appeared in the literature; and to explore the views of clinicians and patients of different metrics for comparing health care provider performance. Different formats for presenting such data to lay and to clinical audiences, which will anchor the choice of metric, have been reported elsewhere [6-8].

## Methods

### Literature review: Frequency of use of different types of metrics

The review sought to identify which metrics had been derived fromeach of the five PROMs used in the National PROMs Programme and what their relative frequency of use has been. The measures include: two generic (EQ-5D and EQ-VAS) [9-11] and three disease-specific PROMs (Aberdeen Varicose Vein Questionnaire (AVVQ) [12]; Oxford Hip Score (OHS) [13]; Oxford Knee Score (OKS) [14]).

As the original conception of these instruments was to measure treatment outcomes, we expected that most of the literature would be evaluations of clinical interventions rather than comparisons of providers. We therefore did not limit our search to the latter use. This ensured that we would capture a wide range of metrics.

We conducted a ‘rapid review’ [15] of the literature using systematic searching and screening methods to produce a sample of literature that would give the range and relative frequency of the metrics of interest. As we were not aiming to provide a comprehensive account of the total number of uses of each metric that had appeared in the literature, a full systematic review was deemed inappropriate.

The searching was conducted across seven databases, hosted by the Ovid interface: EMBASE; Medliner; Global Health; Health Management Information Consortium; PsychInfo; PsychExtra; Social Policy and Practice. The search string combined EQ-5D search terms with a set of terms developed to capture measurement of change and longitudinal studies. Only the EQ-5D required limiting in such a way, as disease-specific PROMs have been used less frequently so were sought using their names only. The full search string was therefore: (EQ-5D terms AND change measurement terms) OR (AVVQ terms or OKS terms or OHS terms).

Expert recommendations for grey literature (i.e. not published in a peer reviewed journal) or for literature currently in press were asked for within our immediate team and teams with overlapping research interests, and were included for screening. Selection was carried out using agreed criteria at title, abstract and full-text screening to capture how the selected PROMs had been applied to measuring change. The primary inclusion criterion was that the paper reported change on at least two occasions, using one of the included PROMs instruments. Full inclusion and exclusion criteria are summarised in Table 1. The following information was extracted from each paper: sample size; number of health care providers; data collection time points; number of outcome measures; methods of determining change in health status.

**Table 1:** Inclusion and exclusion criteria

**Inclusion criteria**	**Exclusion criteria**
Uses one of five PROMs (EQ-5D on its own or supplemented by EuroQol VAS; OHS; OKS; AVVQ) as a study outcome	Focus is not on *using* instruments but assessing if we should use them at all (i.e. review of instruments, assessing psychometric properties, reliability etc.)
Reports change in PROM at two or more time points: At title screen: possible focus on included instrument and measuring change. At abstract screen: focus on included instrument and measuring change. At full-text screen: describes analyses of change using included instrument	Focus is not on included instruments, these must be used as a study outcome (i.e. EQ-5D used in QALY outcomes, and analyses using only EuroQol VAS are excluded)
Peer reviewed and non-peer reviewed literature	Published in foreign language and or no existing English translation

The typology was constructed around two criteria: the type of outcome (a measure of post-intervention health status or the extent of change in health status following an intervention); and whether the metric was based on a continuous variable (eg a mean score) or a categorical variable (eg proportion achieving a defined outcome). Our focus was on identifying frequencies with which these types of metrics were currently used and on generating a shortlist of distinctive metrics appropriate for comparing performance of healthcare providers. Full statistical methods for routine comparisons of performance have been explored elsewhere [16].

Our selections were based on a pre-requisite for such analyses, the potential for complex case-mix adjustment, that is an outcome that could be included as a dependent variable in a statistical model adjusting for the impact of patient characteristics (eg linear or logistic regression to account for socio-demographic, co-morbidities, previous admissions). Metrics that could not be adjusted or were only crudely adjusted (eg stratification) in simple analyses were recorded but deemed unsuitable for our purposes.

Four suitable metrics were selected (all would be adjusted for pre-intervention score) to propose to clinicians and patients:

✓Mean follow-up score

✓Mean change in score

✓Proportion reaching a specified (‘good’) threshold at follow-up

✓Proportion reaching a minimally important difference (MID)

These were chosen to represent the range available from our typology; although we recognise that some measure the same thing (i.e. the mean follow-up and change score would give identical rankings) [17]. And others, if accurately and consistently derived, would likely produce the same or very similar rankings (i.e. using a minimally important difference or a specified threshold for a ‘good’ outcome in the follow-up score).

### Qualitative study: Exploring clinician and patient views on selected metrics

Views of selected metrics were elicited around *accuracy* and *interpretability*. On accuracy we sought views on whether metrics successfully captured differences attributable to provider performance. On interpretability we sought views on the meaningfulness of the proposed metrics.

To obtain such views from surgical clinicians’ meetings were held at six hospitals, supplemented with one meeting at a national conference for clinical staff involved in pre-operative assessment. Meetings took place between September and December 2010. A pragmatic approach to the setting, duration and attendance was adopted given we had to fit in with clinicians’ limited availability. The hospitals were selected from those that had participated in the Patient Outcomes in Surgery (POiS) Audit given that those clinicians had demonstrated an interest in the topic [18]. Seven meetings took place (*N* = 107). Consultants were present at all meetings, nurses or allied health professionals at five meetings, and junior doctors at four meetings (Table 2).

**Table 2:** Description of clinicians who participated in hospital meetings

**Meeting**	**Specialty**	**Type of meeting**	**Profession**			
			**Consultant**	**Junior doctor**	Nurse; AHP*	Other**
A (*N* = 7)	Orthopaedic surgery	Clinical governance	4	3	-	-
B (*N* = 17)	Pre-operative assessment	National conference session	5	-	10	2
C (*N* = 7)	General surgery	Pre-arranged meeting	4	-	1	2
D (*N =* 30)	Orthopaedic surgery	Clinical governance meeting	5	16	9	-
E (*N* = 6)	General surgery; Orthopaedic	Pre-arranged meeting	2	-	4	-
F (*N* = 20)	General surgery; Care of the Elderly	Hospital wide teaching	4	16	-	-
G (*N* = 20)	Orthopaedic surgery	Clinical governance	5	9	4	2
Totals (*N* = 107)	All were surgical units	Tagged to existing agendas or specifically pre-arranged	29	44	28	6

*AHP = Allied Health Professional.

**Managers, administrators, IT staff, clinical audit staff.

To obtain the views of patients, six focus groups were undertaken between October and December 2010, having obtained ethics approval from a Multicentre research Ethics Committee. Arthritis Care (the leading patient organisation for arthritis in the UK) identified 11 participants for the first focus group of whom eight agreed to take part. Participants for the other five groups were selected from those who had taken part in the POiS Audit. Of the 376 people invited, 76 agreed to participate (20%). Selection was stratified by the surgical operation they had undergone, age (54 and under; 55–74; 75 and above), sex and index of multiple deprivation (IMD). This resulted in 45 people attending five meetings; including six partners or lay carers, in response to requests from some patients (Table 3). Consent to participate and for the discussions to be audio-taped was obtained.

**Table 3:** Description of patient and other focus group participants

**Group**	**Sex**		**Operation**				**Age (years)**			Socio- Economic Status (IMD* quintiles)					**Not known**
	M	F	Hip	Knee	VVs	none	40-55	56-75	>75	1	2	3	4	5	-
A *(N = 8)*	3	5	5	2	0	1	2	3	3	2	0	0	0	0	6
B *(N = 7)*	4	3	2	3	0	2	2	3	2	0	2	1	3	0	1
C *(N = 6)*	4	2	0	4	0	2	1	2	3	0	2	2	2	0	0
D *(N = 8)*	4	4	0	8	0	0	2	4	2	3	2	2	1	0	0
E *(N = 9)*	5	4	8	0	0	1	1	4	4	1	2	3	2	1	0
F *(N = 7)*	2	5	0	0	7	0	1	5	1	1	1	4	1	0	0
Totals (*N* = 45)	22	23	15	17	7	6	9	21	15	7	9	12	9	1	7

*Index of multiple deprivation, 1 least deprived to 5 most deprived, or NK = Not known.

VVs = varicose vein surgery.

A &B) London, C) Birmingham, D) Sheffield, E) Liverpool, F) Bournemouth.

**Figure 1:**
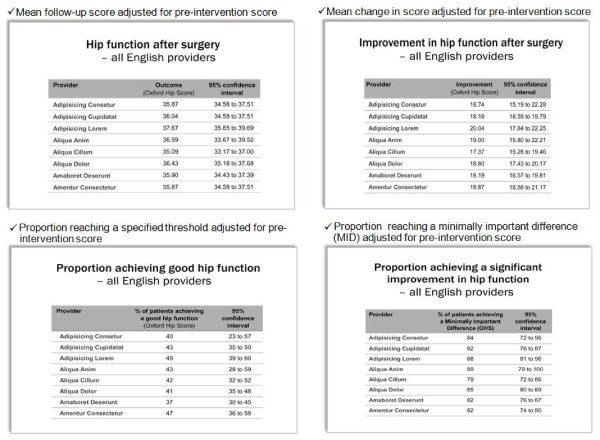
Metrics displays.

Meetings with clinicians and patients were facilitated by one of the authors, accompanied by an observer who took notes. Meetings with clinicians lasted about an hour and with patients about one and a half hours. We sought participants’ views not only of metrics that could be derived from PROMs but also the format and content of how to present data (reported elsewhere [7,8]). To aid the discussions, the Oxford Hip Score was used as the example of a PROM and the four different metrics were explained using this instrument and illustrated in PowerPoint as depicted in Figure 1.

The recordings were transcribed verbatim. Data from clinicians were independently analysed by NB and DA, data from patients by NB and ZH. Thematic analyses (of first and second order concepts) on the views of each metric were summarised with illustrative quotes for each audience. Both sets of findings were then reordered and interpreted (ZH and JN) in a framework analysis that accounted for concepts of accuracy and interpretability which had been identified as two core second order concepts across both audiences. Paired analysis found a high level of inter-rater agreement; where differences occurred, a consensus was achieved through discussion.

## Results

### Literature review

#### Screening and selection

**Figure 2:**
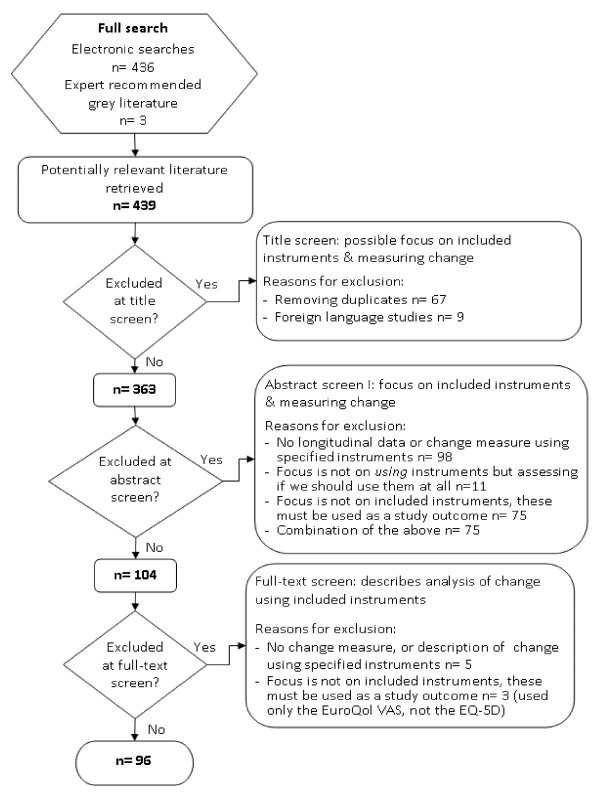
Searching and screening output.

Literature searches carried out in March 2010 identified 439 potential papers. On title screening 67 duplicates and 9 non-English language papers were removed, leaving 363. Of these, 15 cited more than one PROM, thus yielding 388 reported uses of the included measures. Abstract screening led to 72% being excluded because they did not use PROMs as a study outcome or did not report on change in health status. A further seven papers were excluded at full-text screening for the same reasons, leaving 96 papers for analysis (Figure 2).

#### Descriptive information from included studies

Allowing for papers covering more than one measure, the number reporting on each PROM was: EQ-5D 49, EQ-VAS 18, AVVQ 8, OHS 32, OKS 17. About 20% of studies used a single PROM; more commonly several instruments were used, though some which are not included in this review. Sample sizes ranged widely (10 to 9430) with only around 10% having over 1000 cases. The number of time points across which PROMs were collected ranged from two to eight, the mean being two. The length of follow-up for most studies was 6–12 months.

PROMs were used to compare services or health care providers in only six studies [19-24]. Audiences for all the included studies were clinicians, academics or policy-makers. The most frequent use of PROMs was in randomised (n = 29 papers) or non-randomised (n = 41) evaluations of clinical or psycho-social interventions. Other uses were to report differences in outcomes between patient groups (seven papers), variations in outcomes within a specific population (n = 12 studies) or change in people’s health status over time with no intervention (one study).

#### Frequency of use of the different types of metrics

**Figure 3:**
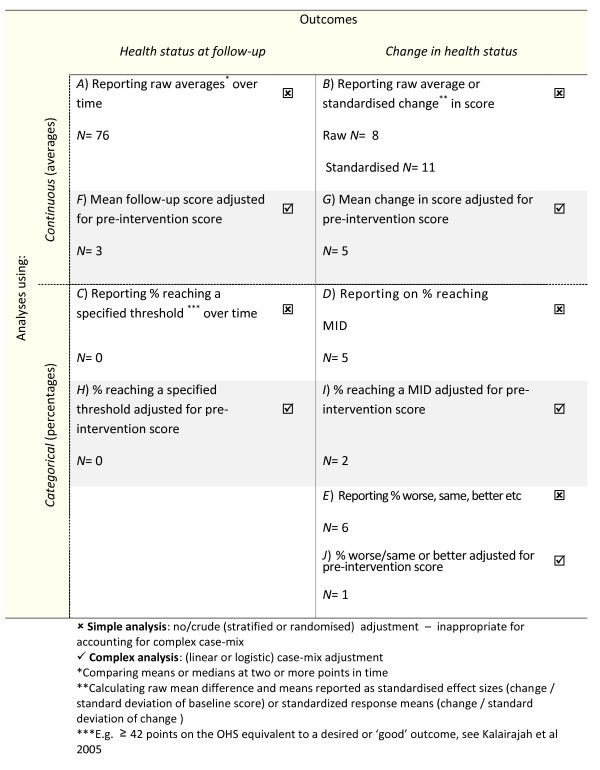
Typology of metrics using before and after PROMs data; highlighted grids were selected for exploring in the clinician meetings and patient focus groups.

In terms of the typology of metrics, see Figure 3*grids A-J*, or the range of anticipated analyses and outcomes, the most frequent metric was simple analyses that reported raw follow-up means or medians, g*rid A*, (n = 76). These averages were compared to pre-intervention averages, implicitly assessing change. Explicit change outcomes in the simple analyses were far fewer, *grid B*, with eight papers reporting mean differences, and 11 using standardised change outcomes (standardised effect sizes or response means). The latter was used for comparing different instruments on consistent scales.

Proportions (categorical outcomes) were also less frequently used in the simple studies. No papers that fitted our inclusion criteria reported on the proportion reporting a specified threshold (e.g.42 points on the OHS as a desired outcome [24]), as represented by *grid C*. Five studies, *grid D*, compared different ways of deriving meaningful benchmarks in change scores based on minimally important differences (MIDs). [24-28].

Earlier studies referred to ‘minimally clinically important difference’ (MCID) [29,30] and ‘minimally clinically important change’ (MCIC) [31]. MIDs can be determined in two ways. The anchor-based method which uses retrospective categorical reports of the success or failure of an intervention (clinician reported initially but more recently patient reported) to determine when a meaningful change occurs. An example of a patient reported anchor would be reporting health as ‘good’ or ‘very good’ after surgery. The distribution-based approach derives the MID by statistical methods, most frequently using half a standard deviation [25,32].

Although the stated long-term aim of the studies was to identify the proportion of patients who achieved a MID and thus help the ‘interpretability’ [31] of the data, most simply evaluated and tested the best method of calculating a MID. A simpler, although perhaps less meaningful way of deriving a proportionate change, was reporting proportions of patients that were worse, the same, or improved (*grid E,* n = 6).

#### Predominance of explicit change outcomes in complex analyses

Only nine studies carried out analysis using complex case-mix adjustment [24,26,27,33-38]. In marked contrast to the simpler analyses, only three studies reported on using a follow-up score, *grid F.* Most used the explicit change outcome in some form. It appears that this outcome may be easier to understand even though the pre-score is double counted. Five complex analyses, *grid G,* used the change score in a linear regression model. No studies specified a threshold for the follow-up score, *grid H*. A couple of studies determining the MID using patient reported anchors, *grid I,* chose to model these in outcomes in logistic regression. Only one paper, *grid J*, considered the proportion improvement, which was reported as arbitrarily selected.

### Clinicians’ and patients’ views on selected metrics

While some patients expressed their views about the different metrics, many found it hard to understand information presented in a quantitative way:

"From my point of view, you’re using a language I don’t understand."

"Ideal for a surgeon or hospital management or whatever but for patients, no."

"To the ordinary man in the street, a lot of its gobbledygook."

In contrast, many clinicians perceived each metric to provide different information to such an extent that they found it difficult to express a preference. They therefore wanted more than one to be made available:

"Why can’t we have all of them? I mean, we’re used to reading papers and understanding data, and they all tell you something slightly different."

"They all look quite important, really. So, I expect if you asked four different clinicians, they’ll say yes to each of them."

Nevertheless, some clinicians were quick to dismiss the options presented to them until they were satisfied that case-mix (characteristics of patients that might affect the outcome of their care such as age, comorbidity and severity) was adequately accounted for, which we were quick to reassure them was part of the analysis:

"What I want is value added. I want it compared to what we started from."

These issues also arose in the patient focus groups, but not explicitly in relation to the different metrics; instead they were expressed in terms of how we might account for some hospitals starting with patients who were sicker than they might be in other hospitals.

Apart from ‘all or nothing’ preferential statements, the differences in the metrics were discussed in terms of their perceived *accuracy* (justified or erroneous) in indicating the performance or quality of providers and their *interpretability*, or perceptions around how easy they were to understand. The clinicians were far more vocal on accuracy than the patients, who cared more about interpretability. Each metric (Mean follow-up; mean change in score; proportion reaching a specified ‘good’ threshold at follow-up; and proportion reaching a minimally important difference (MID) will be considered in turn relative to these concepts.

### Mean follow-up score

#### Accuracy

Clinicians voiced concerns about the accuracy of the mean follow-up score, questioning whether a mean score would be sensitive enough to detecting change over time:

"An improvement in that average might not be possible over quite a few years. Even putting in a great effort, you’ll never show an improvement when you’ve got an average."

A related concern expressed by this audience was that the metric was not transparent enough about the degree of dispersion of individual patient scores to accurately portray performance:

"You’ve got no concept of what your range is…your spread could be enormous or could be really tight. That really matters because you may be making some people worse but if you’re making enough people really good, your mean’s going to be okay."

Patients similarly talked about lack of information on individual variation using this measure, questioning whether this metric may be too subjective to accurately capture performance:

"Everybody’s sense of pain is different, so you can’t say that one person’s average is another person’s good… Some days it’s 20, some days it’s 40 [points]."

This criticism of the metric, also levelled by clinicians at the other continuous metric (change outcome), was interesting because it is no less true of the metrics using categorical outcomes or proportions. Since the unit of analysis is the hospital rather than the patient, outcomes for all proposed metrics would be aggregated at the patient level. Yet both audiences did not think this was a problem with the logistic models presented as proportions.

### Interpretability

Nevertheless, there was support among some clinicians for the interpretability of mean follow-up score. It was thought to be straightforward and likely to influence clinical practice:

"If you present data like this people are going to use it… The main things you’re going to use this for are for the [surgical] department itself to monitor changes within the department."

It was however clear from the discussion surrounding the change score metric (see below), that some clinicians did not recognise that the mean follow-up score was an implicit measure of change; and would give the same results as the explicit measure, given that the pre-intervention score featured in both models.

Patients, on the other hand, did not raise issues to do with explicitly measuring change, but struggled to make sense of what a particular score meant in terms of symptoms and disability:

"Points mean nothing to me… So, before my operation I thought “Where would I like to go to afterwards to get better?” Now, if you could relate from my point of view to my objective, then you’re going somewhere. But if you talk points I’m almost switching off."

These comments were often followed by championing the virtues and universality of the percentage, or a 1–10 scale.

### Mean change in score

#### Accuracy

Many clinicians preferred the mean change to the mean follow-up score. The former was seen as more accurate; since it explicitly took account of the pre-operative score, despite both metrics adjusting for this:

"Change in score is far more useful than an average post-op score…[which] is completely related to where they start and so I don’t believe you could fully take account what it was. So somebody starting with 24 and getting to 48, the fact that they’ve improved by 24 is far more important than…the average [follow-up score]."

The lack of understanding around case-mix adjustment was also seen to render the mean change score potentially *less* accurate as a measure of performance, because it would favour providers whose patients who had greater scope to improve:

"A change in the score is probably going to favour our work because, statistically, our work base is actually older and more severely diseased than most other units."

"It’s a bit like Ryanair being the most improved airline of the year… It’s the baseline level, isn’t it? We need to know what the baseline is before you can assess whether the average change has been important or not."

Another concern from clinicians was whether individual level change was accurately captured. It was not envisaged that clinicians would be misled; instead the problem was projected onto patients:

"What Joe Public won’t know is, is this average change starting from 42 and going to 52 or is it an average change of ten points from 20 points to 30? So in fact, although they’ve got a ten point average change, which suggests it’s a good improvement, they may be going from poor to still less than good, whereas somewhere else might be going from very good to excellent."

Patients however, had little comment to pass of the accuracy of this metric.

### Interpretability

Clinicians, on the whole liked the explicit use of a change metric, finding it meaningful and intuitive, even using the continuous score:

"The change is much more important than a fixed number you’re trying to get people to get to or their point in time score"

Except that, as with the post-operative adjusted metric, patients were unclear what the actual change score meant in terms of improvement in symptoms and disability:

Patient A: Eight points. Eight points of what? If all I felt was 8% better I wouldn’t bother to do it unless I was going to die from it. They don’t mean a thing to me.

Patient B: That’s right, isn’t it, eight points of what?

Patient A: You haven’t told anybody what the points are.

Patients in many ways preferred an outcome to be defined in terms of an experience rather than a number.

### Proportion reaching a specified (‘good’) threshold

#### Accuracy

The accuracy of using the proportion reaching a specified (‘good’) threshold in follow-up score, as a performance indicator, was questioned by clinicians. First, they noted that some patients who would never be able to reach the threshold may still have benefited from surgery:

"[Proportion above threshold] is rubbish, I think, because you don’t know what they start off with… if they come in paralysed from the neck down and you actually enable them to sit up by their own without outside help, then it’s an absolute miracle… You have to know the starting point."

"I did somebody yesterday who’s never going to get to the [threshold] but, actually, considering she started off like this, just getting her sitting up and being able to take five steps with her frame is going to be a huge success for her."

Second, there was concern as to how a threshold for ‘good’ outcome would be determined:

"You’d probably have to be a bit more specific about what [the threshold score] means."

"Can I just say that 42 and over, I think that’s exceptionally high to give a good hip function score. We’ve got a lot of patients who come back with a score of 32, 33, they’re exceptionally happy with the outcome of their surgery."

Although to date clinical appraisal has been used to select the appropriate thresholds [39], it is more fitting that patient reports be used to inform appropriate thresholds in PROMs analyses.

### Interpretability

The proportion reaching a specified threshold, along with the proportion of patients achieving a MID (see below), was perceived by clinicians to be easier for patients, for instance, to interpret over time. The specified threshold was also the metric seen to be the patients favourite:

"These [the proportion over a threshold and proportion with MID] are much more likely to be of benefit to patients because I think they’re easier to understand and you’ll be able to show year-on-year improvement or some solution to a perceived problem much more easily."

"I’d choose that one [proportion over a threshold] because it’s – to me it’s the most important thing that the patients are interested in."

Patients confirmed this, finding it easier to grasp than mean scores:

"The proportion of patients achieving good hip function tells us something about the success and failure of the number of operations that’s being carried out."

"I think that 62% is a good number to latch onto, whereas if you’ve got a point score, peoples’ points out [of however many] are quite different. Whereas if 62% says, “Look, I really feel pretty good”…"

The percentages sign, once again, winning this audience over.

Proportion reaching a minimally important difference (MID)

#### Accuracy

Despite much support, there were also some concerns expressed by clinicians about the accuracy of using a MID as a performance indicator. They suggested that the value of a MID would depend on a patient’s starting point, which was not taken into account:

"[This metric] doesn’t account for non-linearity of the scale. In other words, eight points might be not important at 30 but very important at 40."

They also perceived a potential difficulty in defining what constitutes a MID though assumed it would be decided by clinicians rather than using anchor-based (patient reports) or statistical methods:

"The problem…is you have to establish what’s a minimally acceptable difference. And I suspect we’ll all argue about that. Whereas if you just go for the change we’re achieving, it’s not so debatable."

Their other concern was that, by definition, this metric assessed providers in terms of a minimum standard rather than something more demanding:

"If I, as a patient, had an eight point improvement, well, what does that mean compared to the normal improvement that one would expect from that operation? So am I scoring eight points, when really I should be getting 15."

It was also noted (erroneously) by one clinician that unlike achieving a specified threshold in the follow-up score, using the MID metric would not put providers who operated on more severe patients at a disadvantage:

"They may have a population of people living there who are all 97 and are all crocked or whatever, or start with a very low baseline anyway, that’s just the way they are. You’re still improving their lives by taking them from one to ten."

A lot of assumptions were made about the MID, perhaps because this one of the more complex metrics to derive. Interestingly, this aspect of the measure was not criticised, nor did the clinicians appear phased by explanations of the MID.

### Interpretability

Clinicians argued that despite some drawbacks around accuracy the proportion reaching a MID would be one of the most interpretable metrics, particularly to patients:

"This minimally important difference is clearly what’s important to patients because…it relates to them doesn’t it, not to us? So that would be my favourite one."

"From the patient’s point of view, what they want is an improvement in, presumably, pain and function, so that’s the one that defines that most accurately, I think."

However, some of the support for this metric may have been based on confusing it with the previous one, achieving a threshold:

"The minimally important difference is going to be the threshold, isn’t it? It’s going to be that proportion of patients who can walk with no pain versus those that walk with a bit of pain."

Patients’ interest in this metric was indeed confirmed with the concept of a MID or ‘significant’ improvement being readily interpretable:

"In my case, there was a very significant improvement. I was absolutely hopeless before…I’ve always been really athletically inclined as it were and the people around me are all involved in a club atmosphere, and they noticed that prior I just went sort of down the hill as it were. When I came back I was back up the top of the hill again. So in that respect, the significant difference was that."

"If you knew…a change of five points was not very significant, but a change of eight to ten was more significant… If you knew that before you started, that would be more meaningful."

A criticism often levelled at this metric and anchor based methods of deriving it is that it relies on a single item categorical measure of change, which may itself be imperfect [40]. This issue was not raised by either of the audiences we that we talked to.

## Discussion

### Main findings

The literature review found that the explicit change outcome was the most frequently used. In the audiences of interest to us, both clinicians and patients initially struggled to make a selection when presented with a choice of metrics, though for different reasons. Clinicians wanted variety, as they perceived that most of the metric provided something of value and interest. Patients at times could not distinguish between the four options, but liked a percentage, or what was for them intuitive scaling.

**Figure 4:**
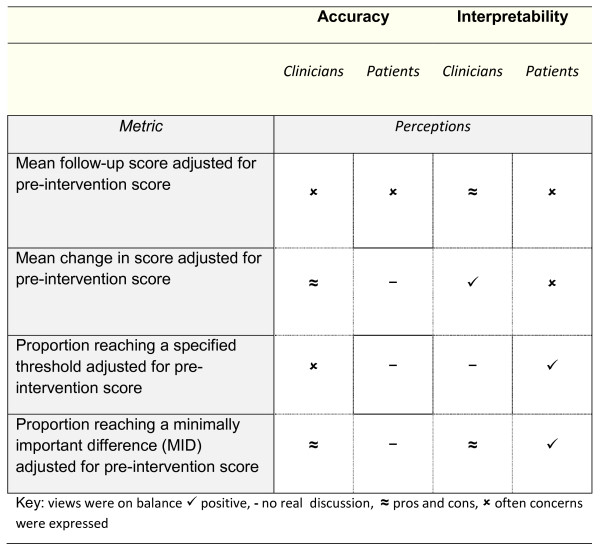
Clinician and patient views of accuracy and interpretability of the four selected metrics.

Metrics were either considered in terms of *interpretability* and or scope of *accuracy* in capturing useful information; views on accuracy did not always match with views on interpretability, and vice versa, see Figure 4. In terms of accuracy, both the metrics based on continuous scores were seen by clinicians as limited in the scope of variation that they allowed to discern; this was not however unique to these metrics, but to the analyses, and applied equally to the categorical or dichotomous metrics. In addition, both patients and clinicians questioned whether continuous outcomes would be able to report actual ‘improvement’ in the quality of care, which was necessarily required to evaluate performance; it was clear from these queries that the adjusted follow-up outcome was not readily identified as an implicit measure of change.

This said some clinicians identified this metric as the most straightforward measure, quickly grasping the scoring; for patients on the other hand, unlike measures of height or weight, PROMs scores are unfamiliar and their values have no immediate meaning. It’s therefore necessary to transform them into interpretable forms, or indeed into experiences rather than metrics, to make them useful. The language used to express the MID seemed to resonate with patients because it captured something of the patient experience. Clinicians echoed the patient endorsement of categories of change, spotting that these that these would be most interpretable to the public.

Categories were seen as easy to understand and sensitive to improvements in performance. However, for clinicians, there were also reservations. They argued that depending on where a threshold was set, the use of this metric might fail to acknowledge the benefit patients would gain from moving from a very severe to a moderately severe condition. In terms of accuracy, therefore the preference was for the using the MIDs despite some concern that this metric only assessed whether a provider was achieving a minimum level of care. The other concerns were methodological: how a threshold or MID would be defined and how non-linearity would be managed.

### Limitations

Given the paucity of literature on comparing provider performance, the review depended on extrapolating from analyses using PROMs in other types of comparison. This nevertheless allowed for consideration of habitual use of these data giving insight into how clinical audiences at least might generally expect to see them used.

For the qualitative studies, we selected participants from those that had volunteered to take part in the earlier POiS Audit. Our sample of surgical clinicians may not, therefore, be representative of the views of all such clinicians but be biased towards those who are more supportive of the use of PROMs. In addition, this study has been limited to the use of PROMs in surgery and, therefore, to the views of surgical clinicians. Clinicians in other areas of health care may hold different views. It was not possible to control the number of attendees, duration and room layout. This made some of the groups quite large and, inevitably, not everyone participated in the discussions.

The heterogeneity of professions and grades that attended the meetings may have affected the views people were prepared to express (e.g. concern about the opinions of peers). It was reassuring that, as regards the medical staff, junior doctors participated as much as their senior colleagues. Although non-medical clinicians attended, they made few contributions such that the majority of the views expressed were those of doctors.

As regards patients, most participants had recently undergone surgery so their views may not therefore represent those of people who are still to make a choice of provider. In addition, their views will have been influenced by their personal experiences of surgery. Whilst representative of patients in terms of age and sex, people from the poorest socio-economic group were under-represented, which may mean that our sample were also more educated.

## Conclusions

Although there was a lack of unanimity both among clinicians and patients as to the best metric policy-makers should choose, a decision has to be made as to which options would be best for dissemination to each audience. Lack of unanimity does not mean there was no discernable consensus. In addition, the discussions helped us to glean how best to explain the metrics in written instructions and labelling, and how to clarify common misunderstandings. These included the use of standard colours, consistent direction of ranking (from best to worst) and clear explanations of concepts such as confidence intervals.

For clinicians, in response to requests for multiple metrics we recommend a categorical and a continuous outcome be used. The mean change in score (adjusted for pre-intervention score) was the most readily grasped. Although some confusion around case-mix would need clarifying, these issues may be easier to address than conveying the implicit change measurement conferred by the mean follow-up metric. Similarly, we recommend using the MID as a dichotomous outcome, and clearly outlining the methodology by which it was derived.

For patients it was apparent that rather than the science behind these measures, the most important aspect of clearly relaying them was using language that would make the metrics personally meaningful and linking these to familiar scaling. For this audience, therefore we propose using the proportion achieving a MID (adjusted for pre-intervention score) or ‘proportion achieving a significant improvement in hip function’.

## Competing interests

The authors declare that they have no competing interests.

## Authors’ contributions

ZH and NB conceived the study; JN, DA and ZH undertook the literature review; DA, ZH and NB undertook the qualitative research and analysis; all authors contributed to drafting the paper. All authors read and approved the final manuscript.

## Pre-publication history

The pre-publication history for this paper can be accessed here:

http://www.biomedcentral.com/1472-6963/12/171/prepub
